# Prognostic factors affecting outcomes in multivisceral en bloc resection for colorectal cancer

**DOI:** 10.6061/clinics/2017(05)01

**Published:** 2017-05

**Authors:** Caio Sergio Rizkallah Nahas, Sergio Carlos Nahas, Ulysses Ribeiro-Junior, Leonardo Bustamante-Lopez, Carlos Frederico Sparapan Marques, Rodrigo Ambar Pinto, Antonio Rocco Imperiale, Guilherme Cutait Cotti, William Carlos Nahas, Daher Cezar Chade, Dariane Sampaio Piato, Fabio Busnardo, Ivan Cecconello

**Affiliations:** I Servico de Cirurgia Gastrointestinal, Instituto do Cancer do Estado de Sao Paulo (ICESP), Hospital das Clinicas HCFMUSP, Faculdade de Medicina, Universidade de Sao Paulo, Sao Paulo, SP, BR; II Servico de Urologia, Instituto do Cancer do Estado de Sao Paulo (ICESP), Hospital das Clinicas HCFMUSP, Faculdade de Medicina, Universidade de Sao Paulo, Sao Paulo, SP, BR; III Servico de Ginecologia, Instituto do Cancer do Estado de Sao Paulo (ICESP), Hospital das Clinicas HCFMUSP, Faculdade de Medicina, Universidade de Sao Paulo, Sao Paulo, SP, BR; IV Servico de Cirurgia Plastica, Instituto do Cancer do Estado de Sao Paulo (ICESP), Hospital das Clinicas HCFMUSP, Faculdade de Medicina, Universidade de Sao Paulo, Sao Paulo, SP, BR

**Keywords:** Colorectal Cancer, Survival, Recurrence

## Abstract

**OBJECTIVES:**

This study sought to determine the clinical and pathological factors associated with perioperative morbidity, mortality and oncological outcomes after multivisceral *en bloc* resection in patients with colorectal cancer.

**METHODS:**

Between January 2009 and February 2014, 105 patients with primary colorectal cancer selected for multivisceral resection were identified from a prospective database. Clinical and pathological factors, perioperative morbidity and mortality and outcomes were obtained from medical records. Estimated local recurrence and overall survival were compared using the log-rank method, and Cox regression analysis was used to determine the independence of the studied parameters. ClinicalTrials.gov: NCT02859155.

**RESULTS:**

The median age of the patients was 60 (range 23-86) years, 66.7% were female, 80% of tumors were located in the rectum, 11.4% had stage-IV disease, and 54.3% received neoadjuvant chemoradiotherapy. The organs most frequently resected were ovaries and annexes (37%). Additionally, 30.5% of patients received abdominoperineal resection. Invasion of other organs was confirmed histologically in 53.5% of patients, and R0 resection was obtained in 72% of patients. The overall morbidity rate of patients in this study was 37.1%. Ureter resection and intraoperative blood transfusion were independently associated with an increased number of complications. The 30-day postoperative mortality rate was 1.9%. After 27 (range 5-57) months of follow-up, the mortality and local recurrence rates were 23% and 15%, respectively. Positive margins were associated with a higher recurrence rate. Positive margins, lymph node involvement, stage III/IV disease, and stage IV disease alone were associated with lower overall survival rates. On multivariate analysis, the only factor associated with reduced survival was lymph node involvement.

**CONCLUSIONS:**

Multivisceral *en bloc* resection for primary colorectal cancer can be performed with acceptable rates of morbidity and mortality and may lead to favorable oncological outcomes.

## INTRODUCTION

Approximately 12% of colorectal cancers (CRC) have adhesions and/or tumoral infiltration into adjacent structures, requiring *en bloc* resection to obtain R0 resection, which is a critical factor for increasing long-term survival 1. Thus, treatment guidelines recommend multivisceral *en bloc* resection (MVEBR) for the treatment of clinically T4b tumors, as most studies have shown that MVEBR improves the rate of R0 resection, which is associated with better local control and overall survival 2,3. Neoadjuvant treatment (especially for rectal cancers) and complete resection, including *en bloc* resection of affected adjacent organs, is the standard clinical recommendation for patients with CRC. Previous studies have reported 5-year survival rates of 36%–53% and local recurrence rates of less than 20% in patients with CRC 2,4,5. In the majority of these studies, achieving an R0 resection was the most important prognostic factor 4,5.

In the majority of cases, T4b CRC patients do not receive MVEBR because the procedure is technically demanding and associated with high perioperative mortality and morbidity when compared to standard colorectal resection 6. MVEBR may also require interactions with other specialist areas, such as urology, plastic surgery, vascular surgery and gynecology, and is quite challenging even for an experienced colorectal surgeon 4. In particular, it is often difficult to intraoperatively distinguish true tumoral invasion from inflammatory adhesions. In fact, tumoral invasion into adjacent structures is confirmed by pathological exam in approximately 50% of cases 3,7.

MVEBR for CRC encompasses a large group of procedures, ranging from simple *en bloc* removal of the colorectal specimen with a small bowel loop to more complex procedures, such as pelvic exenteration in association with resection of sacral bones and large pelvic vessels 3. The clinical and pathological factors associated with morbidity and mortality and oncological outcomes in CRC patients who undergo MVEBR remain a matter of debate.

Therefore, the aim of this study was to determine the clinical and pathological factors associated with perioperative morbidity and mortality and oncological outcomes post-MVEBR in patients with CRC.

## METHODS

Using a prospectively collected database, patients from the Hospital das Clínicas da Faculdade de Medicina da Universidade de São Paulo - Instituto do Câncer do Estado de São Paulo (HCFMUSP-ICESP) with primary (non-recurrent) CRC who required MVEBR between January 2009 and February 2014 were identified and included in the present study. Patients with potentially curative resected distant metastases were also included. Data were obtained after approval from the Institutional Review Board.

Preoperative work-up included colonoscopy and computed tomography of the thorax and abdomen. Pelvic magnetic resonance imaging (MRI) was performed for adequate locoregional staging of rectal tumors. Neoadjuvant chemotherapy (neo CRT) was performed only in patients with lower or mid rectal cancer and not in patients with upper rectal or rectosigmoid junction cancer. Chemotherapy consisted of an intravenous (IV) bolus of 5-FU 350 mg/m^2^ on days 1 to 5 concurrent to radiation in weeks 1 and 5. The total dose of pelvic radiation was 5,040 Gy given in 30 sessions.

Clinical and pathological characteristics included the following: age, gender, tumor location (colon versus rectum), type of surgery (partial colectomy, low anterior resection or abdominoperineal resection), use of neo CRT, number of adjacent structures/organs excised, need for intraoperative blood transfusion, histological differentiation (poor *versus* well differentiated), margins of resection, lymph node involvement, stage of disease, tumor regression grade for rectal tumors treated with neo CRT, and pathological confirmation of invasion of other organs or structures (pT4b).

Information about disease recurrence was obtained from medical records, including medical notes from regular office appointments, endoscopy, radiology, operative and pathology reports. Patients were censored for recurrence at the time of last follow-up. Death was confirmed through system chart review or phone interview with the patient’s family.

### Statistical Analysis

Data were described by estimated sample measures (mean, standard deviation, median, minimum and maximum) for quantitative variables and absolute or relative frequencies for qualitative variables.

The association of complications, death and recurrence with clinical and pathological characteristics in patients without metastasis was assessed using chi-square, Fisher’s exact or likelihood ratio tests. The odds ratios were estimated for each variable of interest with each of the outcomes along with the respective 95% confidence intervals determined from bivariate logistic regression.

Variables from the bivariate tests that showed a level of significance lower than 0.20 (*p*<0.20) were submitted to a multiple logistic regression test, with only variables with a significance level below 0.05 (*p*<0.05) remaining in the final model.

Time of death and recurrence in patients without metastasis were analyzed using the Kaplan-Meier method. Log-rank tests were performed to verify differences in survival time.

The tests were performed with a significance level of *p*<0.05. Data were analyzed using SPSS 20.0 for Windows.

## RESULTS

From January 2009 to February 2014, 1,093 patients with CRC resections were identified at HCFMUSP/ICESP, of which 105 (9.6%) required MVEBR and were included in the present study. Seventy (66.7%) patients were female, and the mean age was 60 years old (range 23-86 years). The mean follow-up period was 27 (6.0-58.7) months. Of the 105 patients, 84 (80%) had primary rectal cancer, and 21 (20%) had primary colon cancer. Most patients had no evidence of metastatic disease at diagnosis (88.6%), and 11.4% received potentially curative resection of liver metastases. Fifty-seven (54.3%) patients with rectal tumors received neo CRT, as this treatment is performed only in patients with lower or mid rectal cancer and not in those with upper rectal and rectosigmoid junction cancers.

All procedures were performed via laparotomy. Low anterior resection was performed in 62% of cases, abdomino-perineal resection in 30.5%, and partial colectomy in 7.5%. Twelve patients had their perineal defect closed by gluteal-fold flaps. Fifty-three patients (50.4%) had only one adjacent organ excised *en bloc* with the colorectal specimen, and 52 patients (49.6%) required excision of two or more adjacent organs. The organs most frequently excised were the ovaries and annexes (37%), followed by the uterus (30%), vagina (26%), bladder (21%), and prostate (13%). The sacrum, small bowel, ureter and other organs were also excised in a few cases. True invasion of adjacent organs was confirmed by way of pathologic assessment in 53.5% of surgical specimens. Free margins were obtained in 81.9% of patients, while complete pathologic response (ypT0N0) was observed in 4.5% of rectal tumors.

Overall, the morbidity rate was 37.1%. Wound infection was the most frequent complication (16.2%), followed by paralytic ileus (8.5%), urinary fistulae (3.8%), intra-abdominal abscess (2.8%), anastomotic leak (1.9%), abdominal wall dehiscence (partial or complete) (1.5%), enteric fistula (1%), and external iliac artery lesion (1%). Ten percent of patients developed significant complications that required surgical intervention. Thirty-two percent of patients received an intraoperative blood transfusion (median 1.7 units, range 1-6). The median length of hospital stay was 11 (range 6-25) days.

Table 1 shows the analysis of potential factors related to complications and the likelihood of these associations. Patients who underwent resection of the ureter or required intraoperative blood transfusion were significantly more likely to suffer a complication (*p*=0.018 and *p*<0.001, respectively). The multiple logistic regression model confirmed that ureter resection and intraoperative blood transfusion were independently associated with a 5.6- and 7.3-fold increased risk of complications, respectively.

The 30-day postoperative mortality rate of 1.9% (2 patients) was not associated with any of the studied parameters. After a mean follow-up period of 27 (range 5-57) months, the overall mortality rate was 23%, and the local recurrence rate was 15%. In non-metastatic patients, a positive margin was the only factor associated with a higher recurrence rate (*p*=0.002).

The Kaplan-Meier curve showed that the estimated 5-year overall survival rate was 47.5%. In univariate analysis, positive margins, lymph node involvement, stage III/IV disease, and stage IV disease alone were associated with lower overall survival, as presented in Table 2. The Cox multiple regression model showed that the presence of lymph node involvement was the only independent risk factor for poor survival.

**Figure 1:**
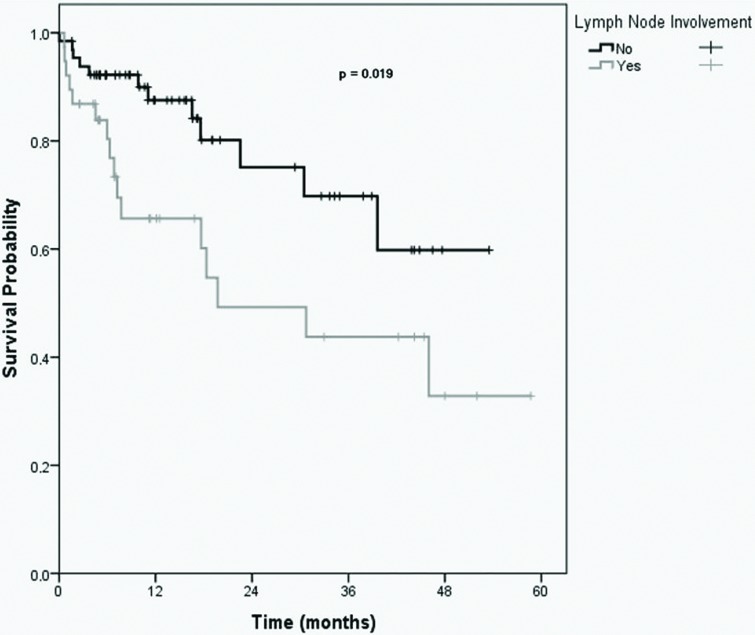
Kaplan-Meier overall survival curves for patients with and without lymph node involvement. (p=0.019).

The estimated 5-year survival rate for patients without metastatic disease was 58.7%, while that for patients with metastatic diseases was 15%. The estimated 5-year survival rates for patients with and without R0 resection were 61.1% and 24.1%, respectively. Finally, the estimated 5-year survival rates for patients with and without lymph node involvement were 32.8% and 59.8%, respectively (Figure 1).

## DISCUSSION

MVEBR for clinically non-recurrent T4b CRC constitutes a demanding group of procedures. However, a multidisciplinary approach can offer favorable oncological outcomes with an acceptable morbidity and mortality rate, as demonstrated in our study.

Direct comparison of our results to those of other studies is difficult due to the wide variation in inclusion criteria (colonic, rectal or both types of tumors), as well as the inclusion of primary and recurrent cancers. In this study, we included only primary advanced CRC patients requiring a multivisceral resection procedure.

In a systematic review of 1,575 patients, 87% of MVEBR cases were performed for non-recurrent CRC, and 64% of the cases had tumors in the rectum 8. In our study, all our cases were non-recurrent with a high proportion of rectal cancer, which is more prone to local invasion of organs and structures in proximity of the tumor.

Reviewing the English-language literature indexed on PubMed between 2008 and 2015, we identified 6 other case series containing a minimum of 40 cases of non-recurrent CRC, as shown in Table 3. Our series represents the second largest number of patients (range of patients included in studies: 42-124). Metastatic patients were present in fewer than 20% of these series. The overall morbidity rate in this study was 37.1%, which is concordant with the results of other series (range 24-50%) 3,9-13. However, it is important to state that only 10% of patients in this study required surgical or image-guided intervention for these complications. Treatment with a multidisciplinary team likely contributed to the positive clinical outcomes in this sample. The use of radiological images (e.g., pelvic MRI images) led to better preparation for surgery and allowed for the organization of a multidisciplinary surgical team to select patients suited to this type of surgery, to plan intraoperative strategies, and to predict difficulties in the postoperative period.

A higher rate of morbidity would be expected in male patients as a result of anatomical characteristics, such as a typically narrower pelvis. However, the two series with predominantly male patients reported morbidity rates of 24% and 50% 3,10. These results were not significantly different from those of studies not composed predominantly of male patients, such as the current study (morbidity ranged from 25-48%) 9,11-13.

Morbidity was higher in cases of tumors located in the rectum and was similar to other studies (39-50%) 3,12,13. Rectal surgery is generally more difficult (especially in narrow pelvises with bulky tumors, and tumors in close proximity to nerves, vessels, and urinary and sexual organs) and carries a greater risk of temporary or definitive stoma. In addition, higher morbidity could also be expected due to the frequent use of neo CRT in rectal tumors.

We did not observe any notable difference in terms of perioperative 30-day mortality between the series (rates varied from 0% to 4%) 3-6. However, this result could not be conclusively demonstrated due to the small sample size.

In the present series, perioperative blood transfusions were associated with a higher frequency of postoperative morbidity, with 7.2 times the overall rate of complications. Few series in the literature have reported the need for blood transfusion, and those that have did not evaluate the correlation with complications. In others studies, 68% of patients required blood transfusions in advanced colonic cancer 10, and 40% of rectal cancer patients required perioperative transfusions 14. Blood transfusion likely reflects the severity of the illness and the more involved nature of the surgery, rather than constituting a risk factor by itself.

Length of stay after a major resection could possibly influence perioperative morbidity. The duration of postoperative hospital stay in the present study was 11 (range 6-25) days. A series of 42 MVEBR procedures for rectal cancer reported that patients stayed a mean of 16 days, which is longer than that reported in this series 3. However, this information was not routinely reported in the other studies 5,9.

In this study, invasion of other organs was confirmed by pathological examination in 53.5% of the samples, which was similar to the majority of the other series (range 34 to 58%) 11. Peritumoral inflammation may result in adhesions that are indistinguishable from true malignant invasion of proximal organs and structures during the surgical procedure. Surgeons must always try to perform *en bloc* resection, including any adherent structures, to achieve R0 resection 15-17. The organs most frequently resected in this study were the ovaries and annexes, followed by the uterus, vagina, bladder, and prostate. Likewise, in the other series that included solely rectal cancer patients, gynecological organs were the organs most frequently resected 10,12,13. The bladder was the most commonly resected organ in some papers, 3 while the small bowel was the most frequently resected in others 9,11. This difference is likely because these two latter series included 66% of patients with colon cancer. In addition, two or more organs were excised in 43% of the procedures in the present study, and this was not significantly associated with complications despite a tendency for this occurrence (*p*=0.058).

Free margins were obtained in 72% of our patients, which is in line with other results in the literature (range 72%-91%) 3,9-13. An R0 resection was identified as a positive prognostic factor in several studies 18-20, while a meta-analysis and single-center study demonstrated significantly reduced overall survival rates for patients in whom a transection of the tumor from adhesive structures was attempted 5,8. In the present series, positive margins increased the recurrence rate and reduced the overall survival rate, which could mean that R0 resection and accurate surgery without excessive tumor manipulation constitute optimal treatment for these patients. A non-R0 resection was also associated with poor prognosis and local recurrence 12 and was associated with poor prognoses in the other four series as well 9,11-13.

Complete pathological response was observed in 4.5% of rectal tumors after neo CRT in this study, similar to the rate of 4.7% reported 3 for rectal tumors in other studies. Assessing the role of neo CRT in cases of complete pathological response was limited by the heterogeneity of studies and the small numbers of included patients. However, patients with a complete pathological response underwent MVEBR, which shows that clinical, radiological and even surgical assessment of the tumor response after neo CRT still has major limitations.

The estimated 5-year overall survival rate of 47.5% is in line with other studies (range 49-69%). In a systematic review, the weighted mean overall 5-year survival rate was 50.3% 8. We would like to emphasize that a 61% survival rate was estimated in patients with R0 resection, comparable to the 64% rate reported by Campos et al. 9. A lower rate of 48% was reported 3 for R0 resection patients.

In this study, lymph node involvement was significantly and independently associated with a decreased survival rate. This association was also observed in another study 13. However, reasonable 5-year survival rates could be achieved with MVEBR even in patients with nodal disease 13,21-24.

Other reported poor prognostic factors for survival included rectal tumor location 9, adverse histological features 9, presence of distant metastasis 3,12, palliative surgery 8,25, and non-sphincter preserving procedures 12.

There are some limitations of this study that need to be acknowledged, including the retrospective collection of data and a short follow-up period.

Ureter resection and intraoperative blood transfusion were associated with higher rates of early postoperative complications. Lymph node involvement was associated with poor overall survival, and positive margins were associated with recurrence in non-metastatic patients.

## AUTHOR CONTRIBUTIONS

Nahas CS, Nahas SC and Ribeiro-Junior U designed the study. Bustamante-Lopez L, Marques CF and Pinto RA analyzed the data. Bustamante-Lopez L and Pinto RA drafted the manuscript. Nahas CS, Nahas SC, Leonardo Bustamante-Lopez L and Pinto RA wrote the manuscript. Nahas CS, Nahas SC and Cecconello I corrected the manuscript. Nahas CS, Marques CF, Pinto RA, Imperiale AR, Cotti GC, Nahas WC, Chade DC, Piato DS and Busnardo F performed the surgeries.

## Figures and Tables

**Table 1:** Description of complications according to the studied variables and tests of association.

	Complication						
Variable	No (N = 66)		Yes (N = 39)		OR	Total (N = 105)	*p*
	N	%	n	%			
**Gender**							>0.999
Female	44	62.9	26	37.1	1.00	70	
Male	22	62.9	13	37.1	1.00	35	
**Primary tumor**							0.106
Rectum	56	66.7	28	33.3	1.00	84	
Colon	10	47.6	11	52.4	2.20	21	
**Type of surgery**							0.926#
APR	21	65.6	11	34.4	1.00	32	
Low anterior resection	40	61.5	25	38.5	1.19	65	
Colectomy	5	62.5	3	37.5	1.15	8	
Gluteal flap**							0.465*
No	12	60	8	40	1.00	20	
Yes	9	75	3	25	0.50	12	
**Bladder**							0.100
No	52	67.5	25	32.5	1.00	77	
Yes	14	50	14	50	2.08	28	
**Uterus**							0.071
No	50	68.5	23	31.5	1.00	73	
Yes	16	50	16	50	2.17	32	
**Vagina**							0.784
No	49	63.6	28	36.4	1.00	77	
Yes	17	60.7	11	39.3	1.13	28	
**Ovaries and annexes**							0.168
No	46	67.6	22	32.4	1.00	68	
Yes	20	54.1	17	45.9	1.78	37	
**Small bowel**							>0.999*
No	58	62.4	35	37.6	1.00	93	
Yes	8	66.7	4	33.3	0.83	12	
**Ureter**							0.018*
No	63	67.0	31	33.0	1.00	94	
Yes	3	27.3	8	72.7	5.42	11	
**Prostate**							>0.999*
No	58	63.0	34	37.0	1.00	92	
Yes	8	61.5	5	38.5	1.07	13	
**Seminal vesicle**							0.758*
No	59	63.4	34	36.6	1.00	93	
Yes	7	58.3	5	41.7	1.24	12	
**Sacrum**							0.293*
No	63	61.8	39	38.2	1.00	102	
Yes	3	100.0	0	0		3	
**Number of organs resected**							0.058
1	38	71.7	15	28.3	1.00	53	
≥2	28	53.8	24	46.2	2.17	52	
**Tumor differentiation**							0.364
Poor	11	73.3	4	26.7	1.00	15	
Well/moderate	55	61.1	35	38.9	1.75	90	
**Blood transfusion**							**<0.001**
No	55	77.5	16	22.5	1.00	71	
Yes	11	32.4	23	67.6	7.19	34	
**Neo CRT**							0.199
No	27	56.2	21	43.8	1.00	48	
Yes	39	68.4	18	31.6	0.59	57	

Result of the chi-square test;

* Result of Fisher’s exact test;

# Result of the likelihood ratio test;

& Is not predictable;

** Neo CRT = Neoadjuvant chemotherapy

APR = Abdominoperineal resection.

**Table 2:** Description of overall survival according to the studied variables.

Variable	Mean time estimate	Std. error (mean)	95% CI	HR	95% CI (HR)	Death by cancer	Total N	%	*p*
**Margins**									**0.027**
Negative	42.6	3.3	36.1-49.0	1.00	1.0-5.1	18	86	20.9	
Positive	25.1	4.8	15.7-34.6	2.35		10	17	58.8	
**N+**									**0.019**
No	40.6	3.2	34.3-46.9	1.00	1.1-5.0	12	65	18.5	
Yes	30.5	4.7	21.2-39.8	2.39		16	38	42.1	
**pT4**									0.129
No	39.6	3.5	32.7-46.5	1.00	0.8-4.1	9	40	22.5	
Yes	34.5	4.1	26.5-42.6	1.84		19	63	30.2	
**Tumor differentiation**									0.252
Poor	29.5	5.4	18.8-40.2	1.00	0.2-1.5	5	14	35.7	
Well or moderate	39.2	3.2	33.0-45.5	0.57		23	89	25.8	
**Blood transfusion**									0.112
No	44.2	3.5	37.4-51.1	1.00	0.8-3.8	14	71	19.7	
Yes	31.0	4.1	23.0-39.1	1.82		14	32	43.8	
**Final stage**									**0.023**
0/I/II	40.3	3.3	33.9-46.8	1.00	1.1-4.9	12	65	18.5	
III/IV	31.2	4.6	22.1-40.3	2.34		16	38	42.1	
**Primary tumor**									0.850
Rectum	35.6	2.7	30.3-41.0	1.00	0.3-2.4	23	83	27.7	
Colon	36.5	7.5	21.9-51.2	0.91		5	20	25.0	
**Type of surgery**									0.821
APR	33.3	3.6	26.3-40.4	1.00		10	31	32.3	
Low anterior resection	39.1	3.8	31.6-46.5	0.78	0.3-1.7	17	64	26.6	
Colectomy	14.5	1.9	10.6-18.4	0.76	0.1-6.0	1	8	12.5	
**Neo CRT**									0.209
No	33.5	4.4	24.8-42.2	1.00	0.3-1.3	16	46	34.8	
Yes	37.9	2.5	32.9-42.9	0.62		12	57	21.1	
**Distant metastases**									**0.018**
No	42.5	3.1	36.3-48.7	1.00	1.1-6.0	20	91	22.0	
Yes	23.1	4.3	14.6-31.7	2.63			8	12	66.7
**Total**	**38.6**	**3.0**	**32.7-44.5**			**28**	103	**27.2**	

Result of the chi-square test; * Result of Fisher’s exact test; #

Neo CRT = Neoadjuvant chemotherapy p: Pathological status N+: Nodal disease

APR = Abdominoperineal resection.

**Table 3:** Summary of the English-language literaturein PubMed regarding multivisceral resection performed in patients with non-recurrent CRC from 2008-2015.

	Present study	Campos 2008	Genzen 2012	Harris 2010	Hoffmann 2012	Smith 2012	Derici 2008
**Number of patients**	105	90	90	42	78	124	57
**Gender M:F**	33%:67%	39%:61%	65%:35%	74%:26%	48%:52%	27%:73%	33%:67%
**Distant metastases**	11%	15%	12%	12%	0%	20%	0%
*R0	**72%**	84.4%%	**91%**	79%	82%	84.6%	75.4%
**True invasion**	53.5%	**57.8%**	**34.4%**	52.3%	48.7%	43%	58%
**N+**	38%	58%	40%	47.7%	**63%**	**25.8%**	61%
**Tumor site**	80% rectal	66% colon	57% colon	100% rectal	66.7% colon	100% rectal	100% rectal
**Organs involved**	Annexes: 37% Uterus: 30% Vagina: 26%	Bowel: 19.8% Bladder: 16.4% Uterus: 12.9%	Ovary: 26.6% Bladder: 25.5% Bowel: 21%	Bladder: 54.7% Uterus/ Annexes: 26.1%	Bowel: 17% Bladder: 10% Ovary: 9.4%	Vagina: 51% Uterus: 23% Bladder: 10%	Annexes: 47% Uterus: 32% Bladder: 30%
**Morbidity**	37.1%	25%	**24.4%**	**50%**	34.6%	47.6%	39%
**Mortality (30 d)**	1.9%	3%	4.2%	**0%**	**7.7%**	0.8%	3.5%
**Overall mortality**	23%	28%	NA	NA	NA	NA	47%
**Local recurrence**	20%	34%	12%	7%	5%	18.8%	18.2%
**5-year survival**	Overall: 47.5% R0: 61% Non-R0: 24% M1: 15%	R0: 64% R1,R2: 0%	Overall 69%	R0: 48% R1,R2: 33% M1: 0%	Overall 55%	Overall 53%	Overall 49%
**Poor prognostic factors for local recurrence**	Resection margins	Tumor location	NR	Metastatic and nodal disease	NR	Resection margins	Adjuvant treatment
**Poor prognostic factors for survival**	Nodal disease	Tumor location	NR	Nodal disease	Resection margins	Resection margins and distant metastases	Nodal disease and resection margins

NR: Not reported.
